# Yindanxinnaotong, a Chinese compound medicine, synergistically attenuates atherosclerosis progress

**DOI:** 10.1038/srep12333

**Published:** 2015-07-21

**Authors:** Long Cheng, Guo-feng Pan, Xiao-dong Zhang, Jian-lu Wang, Wan-dan Wang, Jian-yong Zhang, Hui Wang, Ri-xin Liang, Xiao-bo Sun

**Affiliations:** 1 Key Laboratory of Bioactive Substances and Resources Utilization of Chinese Herbal Medicine, Ministry of Education, Institute of Medicinal Plant Development, Chinese Academy of Medical Sciences and Peking Union Medical College, Beijing, 100193, P. R. China; 2 Department of Traditional Chinese Medicine, Beijing Shijitan Hospital affiliated with Capital Medical University, Beijing, 100038, P. R. China; 3 Center for Drug Evaluation, China Food and Drug Administration, Beijing, 100038, P. R. China; 4 Institute of Medicia Materia, China Academy of Chinese Medical Sciences, Beijing, 100700, P. R. China; 5 Zunyi Medical University, Zunyi, Guizhou 563003, P. R. China; 6 Guang’anmen Hospital, China Academy of Chinese Medical Sciences, Beijing, 100053, P. R. China

## Abstract

Yindanxinnaotong (YD), a traditional Chinese medicine, has been introduced to clinical medicine for more than a decade, while its pharmacological properties are still not to be well addressed. This report aimed to explore the anti-atherosclerosis properties and underlying mechanisms of YD. We initially performed a computational prediction based on a network pharmacology simulation, which clued YD exerted synergistically anti-atherosclerosis properties by vascular endothelium protection, lipid-lowering, anti-inflammation, and anti-oxidation. These outcomes were then validated in atherosclerosis rats. The experiments provided evidences indicating YD’s contribution in this study included, (1) significantly reduced the severity of atherosclerosis, inhibited reconstruction of the artery wall and regulated the lipid profile; (2) enhanced antioxidant power, strengthened the activity of antioxidant enzymes, and decreased malondialdhyde levels; (3) significantly increased the viability of umbilical vein endothelial cells exposed to oxidative stress due to pretreatment with YD; (4) significantly reduced the level of pro-inflammatory cytokines; (5) significantly down-regulated NF-kB/p65 and up-regulated IkB in the YD-treated groups. Overall, these results demonstrated that YD intervention relieves atherosclerosis through regulating lipids, reducing lipid particle deposition in the endothelial layer of artery, enhancing antioxidant power, and repressing inflammation activity by inhibiting the nuclear factor-kappa B signal pathway.

Cardiovascular disease (CVD) is the leading cause of death worldwide, and evidence suggests that half of all CVD cases occur in Asia^1^,^2^. Atherosclerosis (AS), the underlying syndrome of CVD, is a major pathogenic procession involving lipid metabolism, inflammation, innate and adaptive immunity, and many other pathophysiological aspects. Clinical studies have demonstrated that AS may generate a series of cardiovascular events (e.g., acute coronary syndrome and stroke). Secondary to lipid deposition in the vessel wall, AS is a chronic inflammatory disease of arteries and oxidative stress participates in its pathogenesis^2^,^3^,^4^. First and the most critical step in preventing AS involves regulation of the lipid profile. In the early phase of AS, oxidative stress modifies low-density lipoprotein (LDL) to oxidized LDL (ox-LDL), which is taken up by macrophages in the intima of the vascular wall and ultimately leads to foam cell formation.

Another leading cause of AS is inflammation. AS is regarded as a chronic inflammatory disease resulted from the production of cytokines such as interleukin-1β (IL-1β) and IL-10^5^. Interestingly, IL-1β and IL-10 may promote expression of intercellular adhesion molecule-1 and vascular cell adhesion molecule-1 in endothelial cells^6^ as well as interaction between monocytes and endothelial cells, resulting in increased transmigration of circulating monocytes to the intima. Migrated monocytes mature to macrophages, which swallow lipids and become foam cells, leading to inflammatory gene expression and atheromatic plaque formation^7^.

In addition to critically participate in the development of AS, oxidative stress causes endothelial dysfunction, an early feature of AS^8^,^9^,^10^. Thus, the intimate links between lipid deposition, inflammation, and oxidative stress play essential roles in AS. Elucidating the underlying mechanisms of AS may lead to novel prevention and treatment strategies with traditional Chinese medicine (TCM).

Systemic review and meta-analysis suggest that TCM may provide another treatment option for patients with CVD^11^. TCM herbal formulae can be valuable therapeutic strategies and drug resources. Recent reports scientifically verify the clinical benefit of YD^12^,^13^. However, the identification of potent ingredients and their actions are challenges in TCM research. Integrating network biology and polypharmacology promises an expanded opportunity for druggable targets. Network biology may also aid the exploration of drug targets and identify potential active ingredients in TCM research^14^,^15^. The current study used a network pharmacology approach to help determine the active ingredients of YD. We also applied network target prediction and experimental verification to evaluate the links between herbal ingredients and pharmacological actions.

## Results

### YD attenuates atherosclerotic lesions in rats

**Figure 1:**
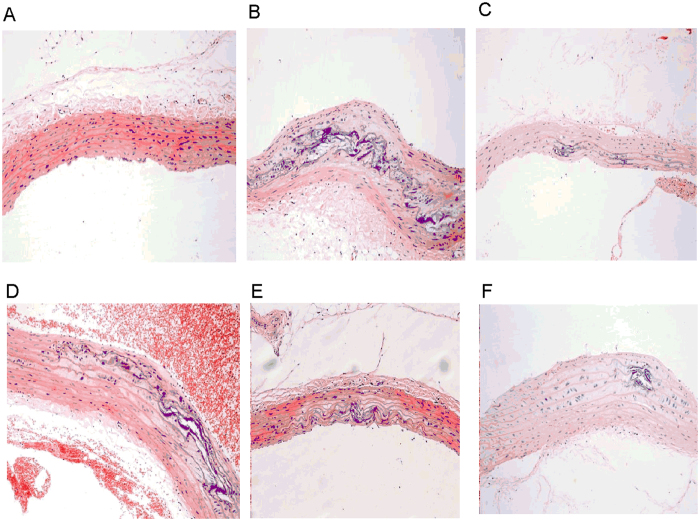
Rat carotid artery sections were subjected to histological examination. Representative photomicrographs of HE staining are shown. Original magnification: ×200. Normal control (**A**) showed no impairment of the artery’s integrity and all layers remained intact, whereas atherosclerotic rats exhibited atherosclerotic lesion formation (**B**). Animals receiving YD (**D**–**F**) and atorvastatin (**C**) intervention showed mild pathological changes compared with normal controls.

After 12 weeks of treatment with YD, light microscopy showed unimpaired integrity and intact layers in hematoxylin-eosin (HE)-stained abdominal aortas from non-atherosclerotic rats (Fig. 1A). In contrast, AS model group (AS group) animals showed thicker and less smooth vessel walls, and the elastic plates in intima and media were damaged (Fig. 1B); we also observed extensive atherosclerotic plaques containing foam cells, inflammatory cells, cholesterol crystals, and tissues calcification(Fig. 1B). Comparing with AS group, pathological changes decreased visibly in YD 0.5 g treated group (YD-0.5), YD 1.0 g treated group (YD-1.0), YD 2.0 g treated group (YD-2.0) (Fig. 1D–F) and atorvastatin treated group (Ator) (Fig. 1C) (i.e., vessel walls were slightly rougher and thicker, and there were fewer atherosclerotic plaques). Notably, both atorvastatin and YD alleviated lipid accumulation and foam cell formation. Comparing with AS group (Fig. 1B), YD intervention (YD-0.5, YD-1.0, YD-2.0 groups) significantly attenuated pathological changes (i.e., slightly rough vessel walls and fewer atherosclerotic plaques).

We measured intima-media thickness (IMT) to quantitatively assess the effect of YD and atorvastatin intervention on the aortic arch. Comparing with normal control group (con group), IMT of the aortic arch was significantly thicker in AS group (90.22 ± 7.78 μm vs. 140.73 ± 16.32 μm, respectively, *P* < 0.01). After 12 weeks of YD and atorvastatin intervention, IMT was significantly thinner (ator 104.83 ± 19.33 μm, YD-2.0 103.37 ± 12.28 μm, YD-1.0 116.27 ± 16.73 μm, YD-0.5 124.77 ± 20.03 μm), comparing with AS group (140.73 ± 16.32 μm).

**Figure 2:**
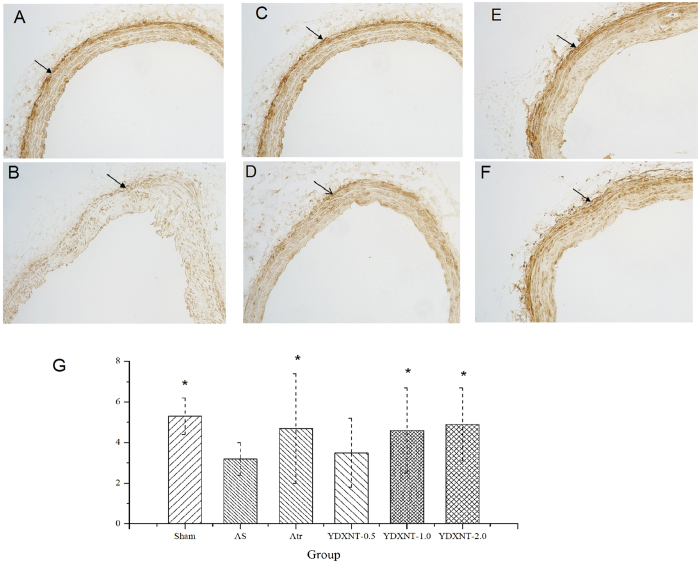
Injecting vitamin D3 & ovalbumin and feeding with high-fat diet induced reconstruction of the carotid artery. Detection of smooth muscle protein 22 alpha (SM22α) expression denotes pathological change in the intima. Compared with the normal-controlled group (**A**), there was low expression of SM22α in intima of atherosclerotic rats (**B**). YD (**D**–**F**) and atorvastatin (**C**) intervention showed relatively high expression of SM22α (**G**). Data denote mean ± SD, n = 12 *P < 0.05 vs. the atherosclerosis group, **P < 0.01 vs. the atherosclerosis group.

Immunohistochemical analysis showed decreased expression of smooth muscle protein 22 alpha (SM22α)^16^,^17^ in intimal smooth muscle cells (SMCs) in AS group (Fig. 2A and Fig. 2B, respectively). Comparing with AS group, atorvastatin or YD treatment yielded significantly augmented expression (Fig. 2G).

### YD regulates the lipid profile in atherosclerotic rats

Comparing with normal-controlled group, cholesterol and TGs increased significantly in AS group; YD reduced such elevation (Table 1). In atherosclerotic rats, YD reduced serum concentrations of total cholesterol, LDL, and TGs. Thus, YD treatment mediated the lipid profile, especially reducing TGs, cholesterol, and LDL. Our data suggest that YD improves the serum lipid profile that associates with the pathogenesis of atherosclerosis. As expected, atorvastatin improved the serum lipid profile as well^16^.

### YD elicits antioxidant action *in vivo*

Table 2 shows the parameters of redox behavior in rats. Compared to AS group, YD significantly (P < 0.05) modified oxidative stress markers after 12 weeks. Malondialdehyde (MDA), an end product of lipid peroxidation, increased significantly in atherosclerotic versus non-atherosclerotic controls.

YD and atorvastatin treatments significantly reduced MDA levels. Concentrations of glutathione (GHS), an antioxidant biomarker, were lower in atherosclerotic rats compared with normal-controlled group, and YD significantly increased GSH levels compared with AS group. Compared with normal-control group, antioxidant enzymes superoxide dismutase (SOD) and selenium-dependent glutathione peroxidase (GSH-px) were significantly less active (*P* < 0.05) in atherosclerotic rats. Moreover, YD treatment yielded significantly higher activity of antioxidant enzymes.

### YD regulates the secretion of pro-inflammatory cytokines and vascular endothelial function markers *in vivo*

AS is a chronic inflammatory disease that results in increased production of inflammatory mediators^18^,^19^,^20^. Our results show that YD and atorvastatin treatment significantly decreased plasma levels of C-reactive protein, tumor necrosis factor alpha (TNF-α), and IL-1β (Table 3), compared with AS group.

Endothelial function is chronically disturbed in atherosclerosis, and endothelial cell dysfunction is a critical step in the progression of the disease^21^,^22^. Our data (Table 4) show significantly decreased concentrations of endothelium-derived relaxing factor (NO) in atherosclerotic rats. Importantly, YD and atorvastatin inhibited such decreases. On the other hand, YD and atorvastatin treatment mediated vasoconstrictor function markers (i.e., endothelin and TXB2).

### Pretreatment with YD attenuated oxidative stress and increased cell viability in HUVECs

To confirm that YD protects against ox-LDL-induced injury, we conducted an MTT assay to check cell viability between groups. Compared with control Group, the ox-LDL-exposed group exhibited significantly decreased cell viability (100% vs. 49%, respectively). We also checked cell viability by pretreating with YD 1.5–100 mg/L. When exposed to ox-LDL-induced oxidative injury, cell viability in groups pretreated with YD (1.5 and 3.0 mg/L) differed little from control group (no pretreated group). Compared with control group, pretreatment with YD (6.25–100 mg/L) significantly increased cell viability (54% ~ 96% *vs.* 49%) after ox-LDL-induced injury. The maximum non-toxicity concentration was 100 mg/L.

### Expression of IkB and NF-kB/p65

**Figure 3:**
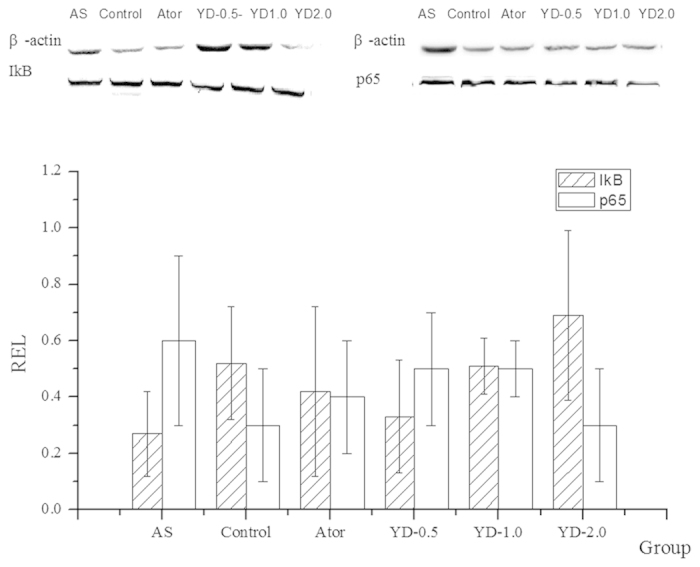
YD pretreatment inhibited NF-kB activation in atherosclerotic rats. YD and atorvastatin intervention increased the average ratios of integral optic density of anti-IkB /β-actin and decreased p65/β-actin.

As shown in Fig. 3, Western blotting revealed increased expression of IkB and significantly decreased levels of nuclear factor-kappa B (NF-kB)/p65 in the YD group compared with AS group. As expected, atorvastatin increased IkB expression and decreased NF-kB/p65 expression (Group Ator).

## Discussion

Network pharmacology offers a new approach of the drug-target exploring and the potential active ingredients identification in TCM research. The present study used network pharmacology to analyze and to predict main drug targets, mechanism of cardiovascular protection. Our experiments verified that YD reduces serum lipids, lowers oxidative stress, and effectively inhibits inflammation in an established rat AS model. To the best of our knowledge, the present study is the first to show that YD attenuates atherosclerosis. Interestingly, atorvastatin and YD offered similar protection in terms of lipid-lowering, anti-inflammation, and anti-oxidation effects.

A previous report showed that high fat diet (HFD) triggers changes in the cardiovascular oxidative state, independent of obesity-induced co-morbidities^23^. We aimed to test the hypothesis that YD can prevent the pathogenesis of AS by improving the blood lipid profile and suppressing oxidative stress and inflammatory processes. Our pathological findings suggest significantly increased plaque formation and lipid deposition in atherosclerotic animals compared with normal controls. YD treatment can alleviate plaque formation and lipid deposition in the artery and can significantly attenuate pro-atherogenic pathological changes.

SM22α—a 22-kDa protein also known as transgelin or WS3-10 and considered a marker of contractile smooth muscle cells (SMCs)—is exclusively and abundantly expressed in SMCs of adult animals. During atherogenesis, SM22-α restricts plaque growth by inhibiting the phenotypic modulation of SMCs from contractile to synthetic/proliferative cells^19^,^20^,^24^. As key pathogenic factor in AS, phenotypic modulation of SMCs affects the recruitment and proliferation of other cells in the lesion. We used immunohistochemistry to elucidate SM22α expression, the inner mechanism that attenuates AS. Our results show that YD treatment inhibited vascular remodeling by increasing SM22-α expression.

CVD is the leading cause of morbidity and mortality worldwide, and sedentary lifestyle and HFD may contribute to increased risk of CVD^1^,^3^. Hyperlipidemia and oxidative stress are among the known risk factors of AS, which is the key and common pathology of CVD. Importantly, recent studies showed that natural and herbal medicines might prevent atherosclerosis by lowering plasma lipid levels and blocking the oxidation of LDL^25^,^26^,^27^. Our data suggest that YD improves the serum lipid profile associated with the pathogenesis of atherosclerosis (Fig. 2). Additionally, YD prevents atherosclerotic lesion development by decreasing TGs and LDL without changing high-density lipoprotein (HDL), yielding a decreased LDL/HDL ratio.

The complex process of atherosclerosis begins when LDL molecules are deposited in the arterial walls and undergo oxidation by reactive oxygen species (ROS) or enzymes such as myeloperoxidase or lipoxygenase. This study focused mainly on elucidating the mechanisms and pathways through which oxidative stress modulates cellular and molecular processes and affects atherosclerotic pathologies. *In vivo*, YD treatment increases the activity of antioxidant enzymes SOD and GSH-px, elevates GSH, and decreases MDA, resulting in significant elevation of total antioxidant capacity. *In vitro*, we used an MTT assay to detect cell viability and confirmed that YD exerted antioxidant activity when exposed to ox-LDL-induced oxidative stress.

Early-stage atherosclerosis associates closely with the inflammatory response of blood vessels that occurs at the beginning of atherosclerotic plaque formation^28^. Thus, an inhibited inflammation response benefits CVD, especially in the early stages of AS. Because transcription factor NF-kB critically induces cytokine-associated genes that participate in the pathogenesis of atherosclerosis^29^,^30^, an activated or inhibited NF-kB signal pathway may play a key role in regulating inflammation and the pathological progression of AS^29^,^31^. *In vivo*, elevated concentrations of plasma inflammation cytokines such as CRP, TNF-α, and IL-1β decreased significantly in atherosclerotic rats undergoing YD intervention. *In vitro,* western blotting showed increased levels of IkB and significantly reduced levels of NF-kB/p65 YD-treated rats. Thus, the anti-inflammation effect of YD may result partly from inhibition of the NF-kB signal pathway.

In summary, our findings demonstrate that YD attenuates AS and inhibits vascular remodeling by reducing wall thickness and the wall thickness-to-diameter ratio. Underlying mechanisms may include regulation of the lipid profile, reduced deposition of lipid particles in the endothelial layer of the artery, enhanced antioxidant power, and anti-inflammation activity via inhibition of NF-kB signal pathway. Our results demonstrate that YD’s ability to inhibit AS may offer a potential therapy for CVD.

## Materials and methods

### Preparation and quality control of YD

In 2002, the China Food and Drug Administration approved YD for the treatment of CVD, and later included YD in the National Essential Medicine List (2012 edition). Guizhou Bailing Pharmaceutical Co., Ltd, provided YD comprised mainly of ginkgo leaves (0.5 g crude drug per capsule), salvia miltiorrhiza (0.5 g crude drug per capsule), herba erigeromtis (0.3 g crude drug per capsule), gynostemma pentaphyllum (0.3 g crude drug per capsule), hawthorn (0.4 g crude drug per capsule), allium sativum (0.4 g crude drug per capsule), panax notoginseng (0.2 g crude drug per capsule), and borneol (0.01 g crude drug per capsule). The principal pharmacologically active components of YD include ginkgo leaves and salvia miltiorrhiza, and its main bioactive constituents include flavonoids (0.59%) and the terpene lactones (0.31%) from ginkgo leaves, salvianolic acid A (0.29%), tanshinone IIA (0.11%), gynostemma total saponins (0.51%), panax notoginseng saponins (5.42%), and borneol (1.21%), which may be responsible for YD’s cardiovascular pharmacological activity. To reduce variation of YD capsules in different batches, Guizhou Bailing strictly standardizes the species, origin, harvest time, medicinal components, and concocted methods for each component. For quality control, Guizhou Bailing uses high-performance liquid chromatography with ultra-violet absorbance optical detector to establish the fingerprint of YD soft capsules (Supplementary Fig. S1).

### Prediction analysis of pharmacological mechanism based on network pharmacology

YD mainly comprises ginkgo leaves, salvia miltiorrhiza, herba erigeromtis, gynostemma pentaphyllum, hawthorn, allium sativum, panax notoginseng and borneol. The active chemical components of YD were collected and extracted from the article database (Chinese Pharmacopoeia 2010 edition, web of science, http://www.wanfangdata.com.cn/, http://www.cnki.net/, www.ncbi.nlm.nih.gov/pubmed/). After reviewing the database, we tried to extract a higher relative content of various medicinal ingredients, taking into account the relevant activity reports. The 173 compounds we collected encompassed the main components of YD.

We put the 173 chemical compounds input TCMSP (http://sm.nwsuaf.edu.cn/lsp/index.php) database to simulate the drug-likeness results by ADME Sico TCMSP database model. Given the integral role of multi-components in TCM, we set at bioavailability (OB) >10%, drug-like >0.04 as the threshold for further extraction and optimization of the medicinal ingredients. To build a compound-medicine network (C-M network), we extracted 63 compounds covering the main drug-likeness component of the YD and put them into the Cytoscape 2.8 software.

**Figure 4:**
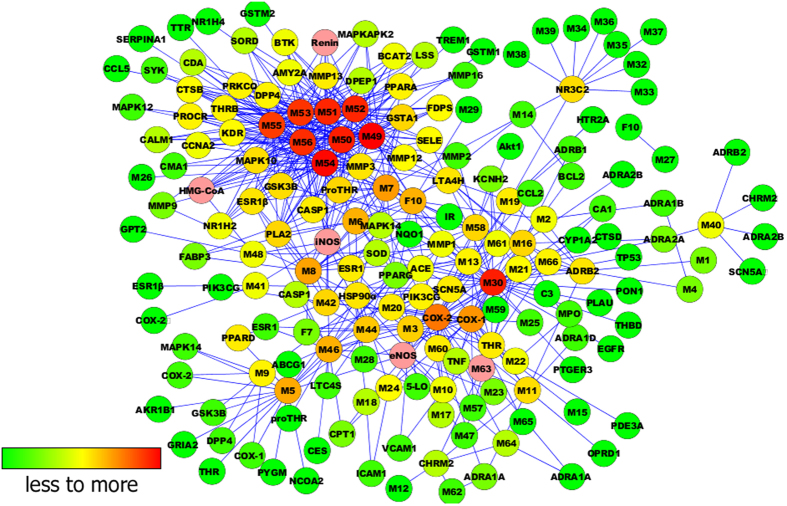
The Compound-Target network.

Next, we used Target-Bank, DrugBank, BindingDB, and PDTD to validate 123 CVD targets. We put the 63 chemical compounds into Cytoscape 2.8 software to build a compound-target network (C-T network, Fig. 4).

**Figure 5:**
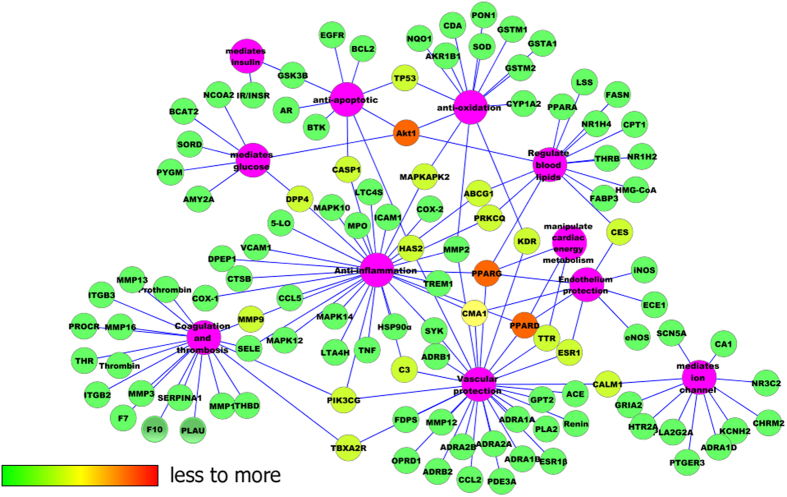
The Target-Function network.

To explain target participation in cardiovascular activity, we used Cytoscape 2.8 to build a network between target and function (T-F network, Fig. 5). Multiple targets indicated an integral role of YD in CVD and cerebrovascular disease, sharing synergy targets of the different compounds.

The main 110 targets included the following functions: anti-inflammation, coagulation and thrombosis, anti-apoptotic, insulin mediation, glucose mediation, cardiac energy metabolism manipulation, ion channel mediation, vascular protection, blood lipids regulation, endothelium protection, and anti-oxidation. To verify this predication, the next experiment was performed accordingly.

### Animal experiments for verification

Sprague Dawley rats (150–200 g) obtained from Beijing Vital River Laboratories Co., Ltd. (Beijing, China) were housed in a controlled environment (temperature 23° ± 2 °C, relative humidity 50% ± 10%, 12 h light/dark cycle) and allowed free access to standard diet. All animals were acclimatized for 5 days prior to the initiation of our experiment. All experimental procedures were conducted in accordance with the Guiding Principles for the Care and Use of Laboratory Animals. The Ethics Committee for Animal Experiments (Institute of Medicinal Plant Development, Chinese Academy of Medical Sciences, Beijing, China) approved our experimental protocol.

We used an AS rat model to evaluate the attenuation of AS and reveal the possible mechanism of YD. Following a procedure established in related reports^31^,^32^,^33^,^34^, we induced AS by injecting the rats with vitamin D3 and ovalbumin and feeding them a high-fat diet (HFD). normal control group rats consumed a regular diet for 21 weeks, whereas rats in AS groups consumed a HFD (1% cholesterol, 0.2% pig bile salts, 10% lard, 10% egg yolk powder, 78.8% basal diet). Initially, rats in the AS model received an intraperitoneal injection of vitamin D3 (600 K IU/kg), followed by a hypodermically injected antigen emulsion (ovalbumin [3 mg/kg] and complete Freund’s adjuvant) to their backs on day 2. After 3 weeks, atherosclerotic rats received weekly intraperitoneal booster injections of ovalbumin (2.5 mg/kg) for 3 consecutive weeks, whereas controls consumed a normal diet and received isovolumic saline injections. At week 9, we randomly divided atherosclerotic rats into five groups for a 12-week treatment regimen, using normal rats as control group (con group). con group and atherosclerotic model controls (AS group) received an equal volume of vehicles. The other atherosclerotic rats received atorvastatin (anti-hyperlipidemics, Ator, 0.01 g/kg b.w., i.g [Ator Group]) or YD treatment (YD 2.0 g/kg b.w. i.g. [YD-2.0 group]); YD 1.0 g/kg BW i.g. [YD-1.0 group]; or YD 0.5 g/kg b.w. i.g. [YD-0.5 group]). Normal control rats (con group) consumed a normal diet throughout the experiment.

After 21 weeks, we deprived all animals of food (but not water) overnight, and then anesthetized them. Next, we drew blood samples from the abdominal aorta, and obtained serum and plasma via blood centrifugation (3,000 rpm for 10 min at 4° C). Prior to histopathologic examination and immunohistochemical analysis, we harvested and weighed the abdominal aorta.

### Histology lesion analysis and immunohistochemical staining

After taking blood samples from the abdominal aorta, we quickly removed the aorta between the aortic valve cusps and the iliac bifurcation, storing them in 4% paraformaldehyde for histological studies. Next, we cut consecutive cross-sections (4 μm thick) of the aorta, and stained with hematoxylin-eosin (HE). Pathological changes were observed with an optical microscope, and intima-media thickness (IMT) was measured using Image-Pro Express 5.0 software.

Immunohistochemical staining were conducted using standard techniques, as described previously^35^. Briefly, we inhibited endogenous peroxidase activity via incubation with 3% H_2_O_2_. After blocking the sections with 5% albumin from bovine serum and incubating them for 10 min, we added primary antibodies and incubated the sections for 60 min before preserving them overnight at 4° C. Following a PBS wash, we incubated the sections with a secondary antibody for 60 min at 37° C. Immunohistochemical staining was visualized using a DAB kit (Boster Biotechnology, LTD, Wuhan, China), according to the manufacturer’s instructions. Immunohistochemical staining (relative positive expression) was quantified using integral optic density

### Biochemical analysis

We collected blood samples and measured serum levels of endothelium-derived relaxing factor (NO), cholesterol, triglycerides (TGs), LDL, and high-density lipoprotein (HDL) using chemical colorimetry assay kits (Nanjing Jiancheng Bioengineering Institute). Rat serum IL-1β, tumor necrosis factor alpha (TNF-α), endothelin, 6-keto-PGF1α, and thromboxane B_2_ (TXB_2_) concentrations were measured by radio-immunity assay, using commercial kits according to manufacturer instructions.

### Measurement of the antioxidative activity *in vivo*

We used total superoxide dismutase (SOD, hydroxylamine method), glutathione peroxidase (GSH-PX, colorimetric method), lipid peroxidation malondialdehyde (MDA), and reduced GSH (spectrophotometric method) assay kits to determine anti-oxidative activity in serum. These commercially available assay kits were provided by Nanjing Jiancheng Bioengineering Institute.

### Culture, proliferation and cytotoxity assay of human umbilical vein endothelial cells

For *in vitro* experiments, we used human umbilical vein endothelial cells (HUVECs) obtained from the Cell Resource Center of the Institute of Basic Medical Sciences, Peking Union Medical College/Chinese Academy of Medical Sciences (Beijing, China), and cultured them in a humidified atmosphere in Dulbecco’s Modified Eagle Medium (DMEM, Thermo US) with 20% fetal bovine serum (20% FBS) at 37° C under 5% CO_2_. The 6th passage cells were used for experiments.

HUVECs (1 × 10^5^) were planted in a 96-well plate, then incubated for 24 h with YD (at the series concentration of 1.5, 3, 6.25, 12.5, 25, 50, 100, 200 mg/L) for protection. After 24 h pretreatment, the supernatant was discarded. Cells then were exposed to 100 mg/L ox-LDL for 24 h to cause oxidative injury, at 37 °C in a 5% CO_2_ atmosphere^10^,^34^,^35^. The viability of HUVEC cells was determined by 3-(4,5-dimethyl thiazol-2-yl)-2,5-diphenyltetrazolium bromide (MTT, sigma) assay to express the protection of YD. The culture medium was replaced with serum-free medium containing 0.5 g/L MTT tetrazolium salt and incubated at 37 °C for 4 hours^36^,^37^. At the end of the incubation with MTT, the cells were scraped and solubilized into DMSO solution. The absorbance was measured at 570 nm by microplate reader (Thermo Scientific). The viability of the cells was expressed as the percentage of the absorbance measured in control cells.

### Western blotting

Using lysis buffer (1%Triton X-100; 50 mmol/L Tris–HCl, pH 7.6; 150 mmol/L NaCl; and 1% protease inhibitor cocktail), we extracted tissue homogenates from the resected left common carotid artery. For western blot, proteins were separated by SDS–PAGE and transferred to polyvinylidene difluoride membranes. After blocking, the membranes were incubated at room temperature for with the indicated antibodies, including anti-p65, anti-IkB, and anti-actin (1:1000 dilution). Next, we treated the membranes with horseradish peroxidase-conjugated secondary antibodies, diluted to 1:2000 (Santa Cruz Biotechnology)^38^,^39^. The blots were developed using ECL western blotting reagents and quantified using optical densitometry. We quantified the bands according to the average ratios of integral optic density of anti-p65/β-actin and anti-IkB/β-actin.

### Statistical analysis

All data are expressed by mean values and standard deviations. Quantitative data were assessed by one-way analysis of variance (ANOVA). If the F distribution was significant, we used a t-test to specify the differences between groups. Differences with a probability value < 0.05 were considered statistically significant. We performed statistical analysis using an SPSS 11.0 software package (SPSS Inc., Chicago, IL, USA).

## Additional Information

**How to cite this article**: Cheng, L. *et al.* Yindanxinnaotong, a Chinese compound medicine, synergistically attenuates atherosclerosis progress. *Sci. Rep.* **5**, 12333; doi: 10.1038/srep12333 (2015).

## Supplementary Material

Supplementary Information

## Figures and Tables

**Table 1:** The serum lipid profile of the YD on atherosclerotic rats (mmol/L).

**Group**	**Dosage (g/kg)**	**CHO**	**LDL**	**HDL**	**TG**
con	—	3.05 ± 0.12**	0.74 ± 0.21**	0.94 ± 0.21	0.41 ± 0.12**
AS	—	7.35 ± 0.43	3.14 ± 0.17	0.69 ± 0.24	1.49 ± 0.40
Ator	0.01	5.82 ± 0.32**	1.91 ± 0.20*	0.91 ± 0.46	0.72 ± 0.32**
YD-2.0	2.0	5.89 ± 0.39**	1.94 ± 0.21*	0.64 ± 0.19	0.79 ± 0.23**
YD-1.0	1.0	5.98 ± 0.42**	2.04 ± 0.24	0.84 ± 0.25	0.88 ± 0.22**
YD-0.5	0.5	6.13 ± 0.40*	2.07 ± 0.11	0.77 ± 0.23	0.93 ± 0.50*

Number of experimental animals (N), total cholesterol (CHO), triacylglycerol (TG), low-density lipoprotein cholesterol (LDL), high-density lipoprotein cholesterol (HDL). Yindanxinnaotong (YD), atorvastatin(Atr).

Results are expressed as mean ± SD, n = 12.

*p < 0.05 *vs* the atherosclerosis model group, **p < 0.01 *vs* the atherosclerosis model group.

**Table 2:** The serum redox parameters of YD on atherosclerotic rats.

**Group**	**Dosage (g/kg)**	**SOD (KU/mL)**	**MDA (mmol/L)**	**GSH (mmol/L)**	**GSH-px (μ mol/L)**
con	—	3.05 ± 0.12**	2.65 ± 0.13**	17.84 ± 2.11**	320.9 ± 21.1**
AS	—	2.16 ± 0.43	5.74 ± 0.22	12.69 ± 1.02	247.0 ± 33.4
Ator	0.01	2.52 ± 0.23**	3.19 ± 0.16**	18.91 ± 0.85**	856.4 ± 19.5**
YD-2.0	2.0	2.69 ± 0.30**	3.34 ± 0.20**	19.64 ± 0.69**	1153.9 ± 30.3**
YD-1.0	1.0	2.78 ± 0.49**	3.64 ± 0.27**	16.84 ± 0.48**	956.8 ± 25.4**
YD-0.5	0.5	2.93 ± 0.42	4.07 ± 0.32**	17.77 ± 1.03**	860.3 ± 30.9**

Number of experimental animals (N), total superoxide dismutase (T-SOD), glutathione peroxidase (GSH-PX), methane dicarboxylic aldehyd (MDA), reduced glutathione (GSH). Yindanxinnaotong (YD), atorvastatin (Ator).

Results are expressed as mean ± SD, n = 12.

*p < 0.05 *vs* the atherosclerosis model group, **p < 0.01 *vs* the athererosclersis model group.

**Table 3:** The effect of YD on serum pro-inflammatory cytokines in atherosclerotic rats.

**Group**	**Dosage (g/kg)**	**TNF-α (ng/L)**	**IL-1β (ng/mL)**	**CRP (ng/mL)**
con	—	20.9 ± 1.1**	0.55 ± 0.03**	5.59 ± 0.43**
AS	—	47.0 ± 3.4	0.74 ± 0.12	8.74 ± 0.72
Ator	0.01	26.4 ± 9.5**	0.59 ± 0.16**	6.19 ± 0.36**
YD-2.0	2.0	33.9 ± 3.3**	0.64 ± 0.10**	5.64 ± 0.50**
YD-1.0	1.0	26.8 ± 5.4**	0.64 ± 0.07**	6.04 ± 0.47**
YD-0.5	0.5	26.3 ± 3.9**	0.67 ± 0.12*	6.07 ± 0.52*

Number of experimental animals (N), interleukin-1β (IL-1β), and tumor necrosis factor alpha (TNF-α). Yindanxinnaotong (YD), atorvastatin(Ator).

Results are expressed as mean ± SD, n = 12.

*p < 0.05 *vs* atherosclerosis model group, **p < 0.01 *vs* the atherosclerosis model group.

**Table 4:** The effect of YD on vascular endothelial function markers in atherosclerotic rats (pg/mL).

**Group**	**Dosage (g/kg)**	**NO (μ mol/L)**	**6-keto-PGF**_**1α**_ **(pg/mL)**	**TXB**_**2**_ **(pg/mL)**	**ET (pg/mL)**
con	—	32.5 ± 1.2**	120.9 ± 21.1	265.1 ± 4.3**	17.8 ± 2.11
AS	—	21.5 ± 2.4	147.0 ± 33.4	344.7 ± 9.2	12.9 ± 1.2
Ator	0.01	28.7 ± 5.3**	156.4 ± 19.5	329.4 ± 10.1*	18.1 ± 8.5
YD-2.0	2.0	26.9 ± 4.3*	153.9 ± 30.3	334.4 ± 7.2*	16.4 ± 6.9
YD-1.0	1.0	25.7 ± 4.9*	156.8 ± 25.4	344.5 ± 20.7	16.8 ± 4.8
YD-0.5	0.5	29.3 ± 5.4**	150.3 ± 30.9	347.3 ± 9.2	17.7 ± 1.3

Number of experimental animals (N), Yindanxinnaotong (YD), atorvastatin (Ator). thromboxane B2(TXB2), endothelin(ET), 6-keto-prostaglandin F1α (6-keto-PGF1α), Nitric oxide(NO).

Results are expressed as mean ± SD, n = 12.

*p < 0.05 *vs* atherosclerosis model group, **p < 0.01 *vs* atherosclerosis model group.

## References

[b1] Ohira T. *et al.* Cardiovascular disease epidemiology in Asia: an overview. Circ J. 77, 1646–52 (2013). 23803294 10.1253/circj.cj-13-0702

[b2] Mendis S., Puska P. & Norrving B. Global atlas on cardiovascular disease prevention and control . 8–13 (World Health Organization, Geneva, 2011).

[b3] Weber C. *et al.* Atherosclerosis: current pathogenesis and therapeutic options. Nat Med. 17, 1410–22 (2011). 22064431 10.1038/nm.2538

[b4] Bleijerveld O. B. *et al.* Proteomics of plaques and novel sources of potential biomarkers for atherosclerosis. Proteomics Clin Appl. 7, 490–503 (2013). 23670906 10.1002/prca.201200119

[b5] Choi J. H. *et al.* Hematein inhibits atherosclerosis by inhibition of reactive oxygen generation and NF-kappa B-dependent inflammatory mediators in hyperlipidemic mice. J Cardiovasc Pharmacol. 42, 287–95 (2003). 12883334 10.1097/00005344-200308000-00019

[b6] Lee G. *et al.* 4-O-methylgallic acid down-regulates endothelial adhesion molecule expression by inhibiting NF-kappaB-DNA-binding activity. Eur J Pharmacol. 551, 143–51 (2006). 17027748 10.1016/j.ejphar.2006.08.061

[b7] Libby P., Ridker P. M. & Hansson G. K. Inflammation in atherosclerosis: from pathophysiology to practice. J Am Coll Cardiol. 54, 2129–38 (2009). 19942084 10.1016/j.jacc.2009.09.009 PMC2834169

[b8] Davignon J. *et al.* Role of endothelial dysfunction in atherosclerosis. Circulation . 109, III27–32 (2004). 15198963 10.1161/01.CIR.0000131515.03336.f8

[b9] Libby P. *et al.* Progress and challenges in translating the biology of atherosclerosis. Nature . 473, 317–25 (2011). 21593864 10.1038/nature10146

[b10] Chen X. P. *et al.* Oxidized low density lipoprotein receptor-1 mediates oxidized low density lipoprotein-induced apoptosis in human umbilical vein endothelial cells: role of reactive oxygen species. Vascul Pharmacolo. 7, 1–97 (2004). 10.1016/j.vph.2007.01.004 17433786

[b11] Wang J. Y. *et al.* Potential effectiveness of traditional Chinese medicine for cardiac syndrome X (CSX): a systematic review and meta-analysis. BMC Complement Altern Med. 13, 62 (2013). 23497135 10.1186/1472-6882-13-62 PMC3662595

[b12] Wang W. *et al.* Protective effects of yindanxinnaotong capsule in a rat model of myocardial ischemia/reperfusion injury. J Tradit Chin Med. 34, 699–709 (2014). 25618975 10.1016/s0254-6272(15)30085-6

[b13] Wang W. *et al.* Protection of yindan xinnao tong capsule and main compositions compatibility on myocardial ischemia/reperfusion injury Zhongguo Zhong Yao Za Zhi. 39, 1690–4 (2014). In Chinese. 25095386

[b14] Hopkins A. L. *et al.* Network pharmacology. Nat Biotechnol. 25, 1110–1 (2007). 17921993 10.1038/nbt1007-1110

[b15] Li S. *et al.* Traditional Chinese medicine network pharmacology: theory, methodology and application. Chin J Nat Med. 11, 110–20 (2013). 23787177 10.1016/S1875-5364(13)60037-0

[b16] Park J. H. *et al.* The clinical significance of the atrial subendocardial smooth muscle layer and cardiac myofibroblasts in human atrial tissue with valvular atrial fibrillation. Cardiovasc Pathol. 22, 58–64 (2013). 22658273 10.1016/j.carpath.2012.05.001

[b17] Han M. *et al.* Smooth muscle 22 alpha maintains the differentiated phenotype of vascular smooth muscle cells by inducing filamentous actin bundling. Life Sci. 84, 394–401 (2009). 19073196 10.1016/j.lfs.2008.11.017

[b18] Jamkhande P. G. *et al.* Therapeutic approaches to drug targets in atherosclerosis. Saudi Pharm J. 22, 179–90 (2014). 25061401 10.1016/j.jsps.2013.04.005 PMC4099571

[b19] Kalz J. *et al.* Thrombin generation and atherosclerosis. J Thromb Thrombolysis . 37, 45–55 (2014). 24241912 10.1007/s11239-013-1026-5

[b20] Wolak T. Osteopontin - A multi-modal marker and mediator in atherosclerotic vascular disease. Atherosclerosis . 236, 327–37 (2014). 25128758 10.1016/j.atherosclerosis.2014.07.004

[b21] Lee P. S. *et al.* Endothelial progenitor cells in cardiovascular diseases. World J Stem Cells . 6, 355–66 (2014). 25126384 10.4252/wjsc.v6.i3.355 PMC4131276

[b22] Shaw A. *et al.* Endothelial cell oxidative stress in diabetes: a key driver of cardiovascular complications. Biochem Soc Trans. 42, 928–33 (2014). 25109981 10.1042/BST20140113

[b23] Chalkiadaki A. *et al.* High-fat diet triggers inflammation-induced cleavage of SIRT1 in adipose tissue to promote metabolic dysfunction. Cell Metab. 16, 180–8 (2012). 22883230 10.1016/j.cmet.2012.07.003 PMC3539750

[b24] Feil S. *et al.* SM22alpha modulates vascular smooth muscle cell phenotype during atherogenesis. Circ Res. 94, 863–5 (2004). 15044321 10.1161/01.RES.0000126417.38728.F6

[b25] Zhang J. Y. *et al.* Effects of an aqueous extract of Crataegus pinnatifida Bge. var. major N.E.Br. fruit on experimental atherosclerosis in rats. J Ethnopharmacol. 148, 563–9 (2013). 23685195 10.1016/j.jep.2013.04.053

[b26] Su W. *et al.* Tongxinluo inhibits oxidized low-density lipoprotein-induced maturation of human dendritic cells via activating peroxisome proliferator-activated receptor gamma pathway. J Cardiovasc Pharmacol. 56, 177–83 (2010). 20489656 10.1097/FJC.0b013e3181e5f0f8

[b27] Cheng L. *et al.* Evaluation of anxiolytic-like effect of aqueous extract of asparagus stem in mice. Evid Based Complement Alternat Med. 2013, 587260 (2013). 24348707 10.1155/2013/587260 PMC3853311

[b28] Ross R. Atherosclerosis—an inflammatory disease. N Engl J Med. 340, 115–26 (1999). 9887164 10.1056/NEJM199901143400207

[b29] Choi J. H. *et al.* Hematein inhibits atherosclerosis by inhibition of reactive oxygen generation and NF-kappaB-dependent inflammatory mediators in hyperlipidemic mice. J Cardiovasc Pharmacol. 42, 287–95(2003). 12883334 10.1097/00005344-200308000-00019

[b30] Kim J. H. *et al.* Functional dissection of Nrf2-dependent phase II genes in vascular inflammation and endotoxic injury using Keap1 siRNA. Free Radic Biol Med. 53, 629–40 (2012). 22609006 10.1016/j.freeradbiomed.2012.04.019

[b31] Gocmen A. Y. *et al.* Effect of atorvastatin on atherosclerotic plaque formation and platelet activation in hypercholesterolemic rats. Can J Physiol Pharmacol. 91, 680–5 (2013). 23984971 10.1139/cjpp-2012-0325

[b32] Li Y. *et al.* Rosuvastatin attenuates atherosclerosis in rats via activation of scavenger receptor class B type I. Eur J Pharmacol. 723, 23–8 (2014). 24333476 10.1016/j.ejphar.2013.11.037

[b33] Zhou B. R. *et al.* Fibrinogen facilitates atherosclerotic formation in Sprague-Dawley rats: A rodent model of atherosclerosis. Exp Ther Med. 5, 730–34 (2013). 23408727 10.3892/etm.2013.913 PMC3570179

[b34] Xu X. *et al.* Amelioration of lipid profile and level of aAntioxidant activities by epigallocatechin-gallate in a rat model of atherogenesis. Heart Lung Circ. 23, 1194–201 (2014). 25027849 10.1016/j.hlc.2014.05.013

[b35] Torzewski M. *et al.* Immunohistochemical demonstration of enzymatically modified human LDL and its colocalization with the terminal complement complex in the early atherosclerotic lesion. Arterioscler Thromb Vasc Biol. 18, 369–78 (1998). 9514405 10.1161/01.atv.18.3.369

[b36] Guo H. *et al.* Resveratrol protects HUVECs from oxidized-LDL induced oxidative damage by autophagy upregulation via the AMPK/SIRT1 pathway. Cardiovasc Drugs Ther. 27, 189–98 (2013). 23358928 10.1007/s10557-013-6442-4

[b37] Huang C. S. *et al.* Isothiocyanates protect against oxidized LDL-induced endothelial dysfunction by upregulating Nrf2-dependentantioxidation and suppressing NF-κB activation. Mol Nutr Food Res. 57, 1918–30 (2013). 23836589 10.1002/mnfr.201300063

[b38] Gareus R. Endothelial cell-specific NF-kappaB inhibition protects mice from atherosclerosis. Cell Metab. 8, 372–83 (2008). 19046569 10.1016/j.cmet.2008.08.016

[b39] Matsumoto T. *et al.* Local elastic modulus of atherosclerotic lesions of rabbit thoracic aortas measured by pipette aspiration method. Physiol Meas. 23, 635–38 (2002). 12450265 10.1088/0967-3334/23/4/304

