# Supplementary material for: Yindanxinnaotong, a Chinese compound medicine, synergistically attenuates atherosclerosis progress
Source: Sci Rep. 2015 Jul 21;5:12333. doi: 10.1038/srep12333 (PMC4508829; doi:10.1038/srep12333)

Supplementary File

Supplementary Figure. 1 the fingerprint chromatography of Yindanxinnaotong

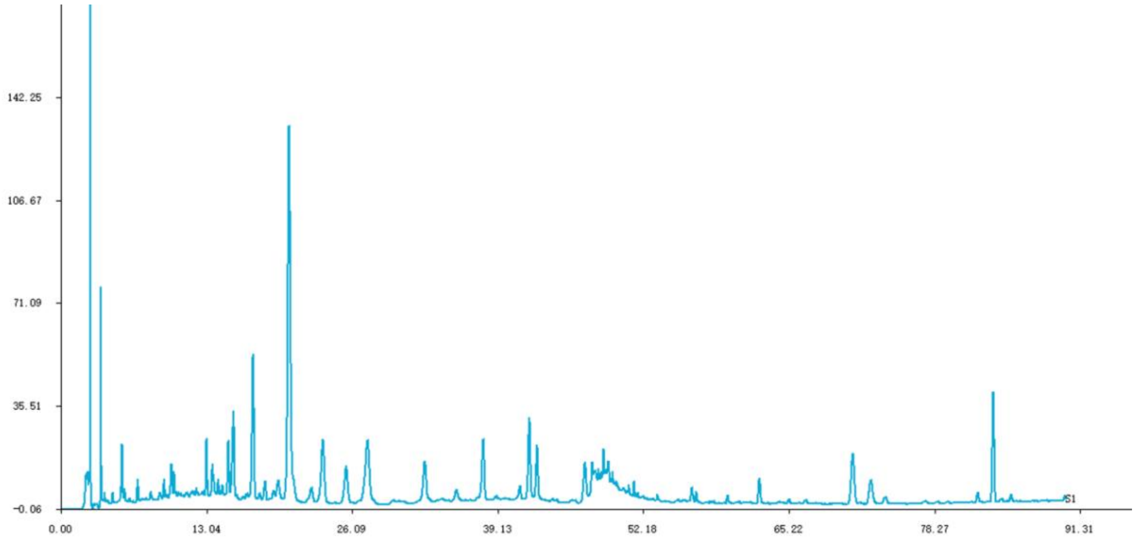

Supplement: Supplementary Information [file srep12333-s1.pdf]
